# Membrane Potential Generated by Ion Adsorption

**DOI:** 10.3390/membranes4020257

**Published:** 2014-06-12

**Authors:** Hirohisa Tamagawa, Sachi Morita

**Affiliations:** Department of Human and Information Systems, Faculty of Engineering, Gifu University, 1-1 Yanagido, Gifu 501-1193, Japan; E-Mail: tenjoinfifty@gmail.com

**Keywords:** membrane potential, Goldman-Hodgkin-Katz equation, ion permeability, ion adsorption, Langmuir isotherm, Boltzmann distribution, Poisson-Boltzmann equation

## Abstract

It has been widely acknowledged that the Goldman-Hodgkin-Katz (GHK) equation fully explains membrane potential behavior. The fundamental facet of the GHK equation lies in its consideration of permeability of membrane to ions, when the membrane serves as a separator for separating two electrolytic solutions. The GHK equation describes that: variation of membrane permeability to ion in accordance with ion species results in the variation of the membrane potential. However, nonzero potential was observed even across the impermeable membrane (or separator) separating two electrolytic solutions. It gave rise to a question concerning the validity of the GHK equation for explaining the membrane potential generation. In this work, an alternative theory was proposed. It is the adsorption theory. The adsorption theory attributes the membrane potential generation to the ion adsorption onto the membrane (or separator) surface not to the ion passage through the membrane (or separator). The computationally obtained potential behavior based on the adsorption theory was in good agreement with the experimentally observed potential whether the membrane (or separator) was permeable to ions or not. It was strongly speculated that the membrane potential origin could lie primarily in the ion adsorption on the membrane (or separator) rather than the membrane permeability to ions. It might be necessary to reconsider the origin of membrane potential which has been so far believed explicable by the GHK equation.

## 1. Introduction

Potential generation between two electrolytic solutions separated with an ion-permeable membrane is one of fundamental phenomena in biology field known as membrane potential. Membrane potential has been for decades studied by a number of researchers [1,2,3,4]. It is widely accepted at present that the Goldman-Hodgkin-Katz (GHK) equation fully explains the behavior of membrane potential. The GHK equation explains that membrane permeability varies in accordance with ion species in the aqueous solution, and it is one of fundamental factors determining how high potential is generated across the membrane. Cell potential across the cell membrane is of course a membrane potential, and its behavior is well-explained by the GHK equation. The cell membrane potential often exhibits abrupt changes or even more complex behavior, but it can be well explained by the GHK equation. According to the GHK equation, the complex change of membrane permeability to the ions results in the complex cell membrane potential behavior. Hence, basically, membrane permeability is a dominant factor for cell membrane potential generation. On the other hand, it has been continuously reported the existence of the membrane potential inexplicable by the GHK equation by a small number of researchers [1,2]. Colacicco measured that potential across an oil membrane separating two electrolytic solutions [2,5,6]. He observed nonzero potential despite the impermeability of oil membrane to the ions. His observation completely contradicts the prediction by the GHK equation. Not only Colacicco’s experiment, the detailed literature survey reveals that there have been a number of other reports contradicting the GHK equation (in other words the current membrane theory) for decades up until today [1,2].

The leading scientist at present who challenges the current membrane theory is a physiologist Dr. Gilbert Ling. He has advocated his own theory for explaining the origin of the membrane potential. Ling insists that the membrane permeability to the ions has nothing to do with the potential generation and the ions adsorption on the membrane surface generates the membrane potential. His theory is in harmony with all the past reports about the membrane potential generation, and even the reports which have been explicable by the GHK equation are within the range of his theory [1,2]. According to his masterpieces of references [1,2], derivation procedure of GHK equation was given by Goldman, but the current physiological meaning of the GHK equation was given by Hodgkin and Katz. Namely, Hodgkin and Katz are the actual originators of GHK equation in sense of current physiology. However, a number of scientists even including those originators faced experimental evidences contradicting the prediction based on the GHK equation [1,2,7,8].

An electrochemist, Dr. Cheng, decades ago proposed a similar theory to the Ling’s for explaining the potential generation of the glass electrode [9,10,11,12,13]. Glass electrode exhibits potential generation in accordance with proton concentration surrounding it. Cheng concludes that the potential generation is due to the ion adsorption on the glass membrane surface. Ling and Cheng reached a fairly similar conclusion independently from each other concerning the mechanism of potential generation across the membrane.

Our study of membrane potential that will be described in this paper also brought us a conclusion that the membrane potential might have almost nothing to do with the membrane permeability to the ions, and the ion adsorption on the membrane surface appears to have dominant influence on the potential generation. Based on our experimental observations, we derived an equation for explaining the potential we experimentally observed. The equation never takes into consideration the passage of ions through the membrane but needs to take into consideration the ion adsorption on the membrane surface. The computational potential behavior obtained by employing the equation was in good agreement with the experimental results.

## 2. Experimental Section

### 2.1. Potential across the Anion Exchange Membrane

**Figure 1:**
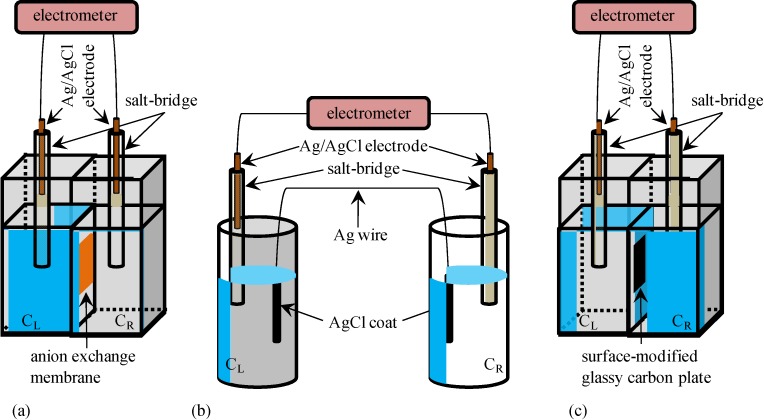
Experimental setup for measuring the potential between two KCl aqueous solutions separated through the intermediary of (**a**) a anion exchange membrane; (**b**) an Ag wire; and (**c**) a surface-modified glassy carbon plate KCl concentrations in the left and right compartments are represented by *C*_L_ and *C*_R_, respectively, for all the setups.

Potential generated between two KCl aqueous solutions was measured using an electrometer of HE-104A (HOKUTO DENKO CO., Tokyo, Japan) and Ag/AgCl electrodes. All the KCl solution used in this research were prepared by dissolving KCl with highly deionized water. These two KCl solutions were in contact with each other through the intermediary of a sheet of Selemion AMV (Asahi Glass Co., Ltd., Tokyo, Japan) which is an anion exchange membrane. The anion exchange membrane is quite permeable to anions and less permeable to cations, since it contains fixed cations and mobile anions in the hydrated state. Whole experimental system is illustrated in Figure 1a. KCl concentration of left solution is represented by *C*_L_ while that of right solution is represented by *C*_R_.

### 2.2. Potential across the Ag Wire Coated with AgCl

**Figure 2:**
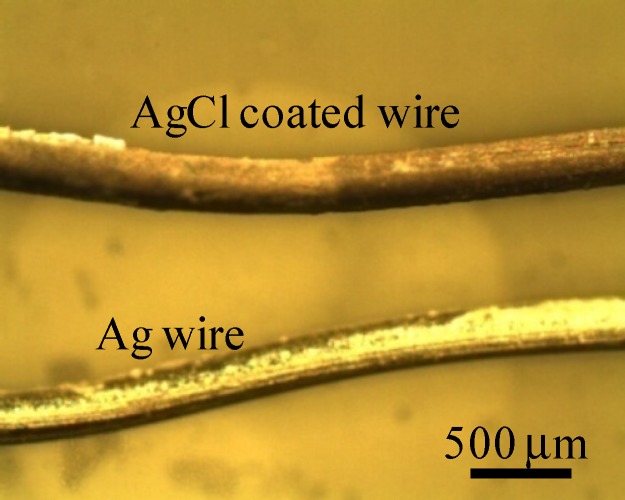
Photo of an Ag wire and an Ag wire coated with AgCl.

Potential generated between two separately placed KCl aqueous solutions was measured using a setup illustrated in Figure 1b. These two KCl solutions were electrically in contact with each other through the intermediary of an Ag wire both ends of which were coated with AgCl. This wire was, of course, impermeable to ions. AgCl coating on the ends of Ag wire was carried out simply by submerging these ends in chlorine bleach for 1 h. Figure 2 shows a photo of an Ag wire and an Ag wire coated with AgCl.

### 2.3. Potential across the Glassy Carbon Plate Covered with –COOH Groups

Potential generated between two KCl aqueous solutions separated with a surface-modified glassy carbon plate was measured as illustrated in Figure 1c. Since the glassy carbon is an electrically conductive material, the two KCl solutions were electrically in contact with each other, though the glassy carbon plate is completely impermeable to ions.

The surface-modified glassy carbon plate we use had –COOH groups on its surface. Its fabrication procedure is explained here. A glassy carbon plate was placed in mixed acid consisting of HNO_3_ and H_2_SO_4_ weight ratio 1:3 [14,15]. This process creates –COOH groups on the surface of glassy carbon. Three types of surface-modified glassy carbon plates were prepared and they are designated as GC1, GC2 and GC3 (see below), respectively.

GC1: A glassy carbon plate was placed in a mixed acid for 8 h at 302 K. After this treatment, the glassy carbon plate was washed with deionized water and dried in atmosphere.

GC2: A glassy carbon plate was placed in a mixed acid for 36 h at 302 K. After this treatment, the glassy carbon plate was washed with deionized water and dried in atmosphere.

GC3: A glassy carbon plate was placed in a mixed acid for 36 h at 318 K in hot water. After this treatment, the glassy carbon plate was washed with deionized water and dried in atmosphere.

From the view point of chemistry, the concentration of –COOH on the surface of the glassy carbon plates increases in the order of GC1–GC2–GC3.

## 3. Results and Theoretical Analysis

We made measurement of potential, *V*, generated between two KCl solutions. In this paper, *V* is defined as the potential of KCl solution in the left compartment in reference to that in the right compartment.

### 3.1. Potential Generation across the Anion Exchange Membrane

Potential across the anion exchange membrane separating two KCl solutions was measured at the environmental temperature *T* = 291 K using the setup Figure 1a described in the Section 2.1. KCl concentration of *C*_L_ was increased from 10^−5^ M to 3.4 M, while that of *C*_R_ was maintained constant at 0.1 M. According to the current membrane theory, the membrane potential is given by Equation (1), which is based on the GHK Equation.
*V* = 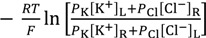
(1)
where *P*_K_ and *P*_Cl_ represent the permeability of membrane to K^+^ and Cl^−^, respectively; [K^+^]_L_ and [K^+^]_R_ represent the K^+^ concentration in the left and right compartments, respectively; and [Cl^−^]_L_ and [Cl^−^]_R_ represent the Cl^−^ concentration in the left and right compartments, respectively; *R* and *F* represent gas constant and Faraday constant, respectively.

Assuming the anion exchange membrane is virtually impermeable to the cation, K^+^, Equation (1) is reduced into Equation (2).
*V* = 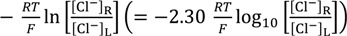
(2)
Namely, *V* is a function of merely the ratio [Cl^−^]_R_/[Cl^−^]_L_. Since [Cl^−^]_R_ = *C*_R_ and [Cl^−^]_L_ = *C*_L_, [Cl^−^]_R_/[Cl^−^]_L_ = *C*_R_/*C*_L_.

**Figure 3:**
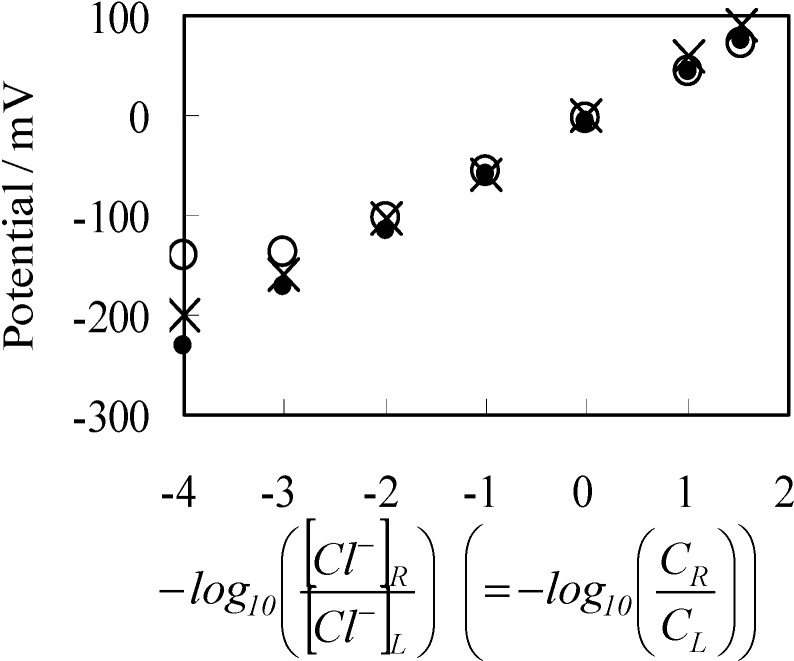
Potential generated between two KCl solutions *vs.* −log([Cl^−^]_R_/[Cl^−^]_L_). ○: an anion exchange membrane separator; ×: Ag wire separator; •: potential computed by employing the Goldman-Hodgkin-Katz (GHK) equation.

Figure 3 shows the experimentally observed potential (○ mark) and the computational potential data (• mark) obtained by using Equation (2), where horizontal axis represents −log_10_([Cl^−^]_R_/[Cl^−^]_L_). The computational potential data was obtained by considering ion activity of KCl [16], while the its horizontal axis quantities were all computed under the assumption that the activity coefficients were 1 so that all the potential data in Figure 3 can be directly compared one another.

Experimentally observed potential was obtained, once the potential reached the stable potential, and usually such a stable potential was achieved tens seconds after supplying two KCl solutions in both left and right compartments of setup illustrated in Figure 1a. Measurements were made a number of times in order to confirm the data reproducibility of potential. So the experimental data represented by ○ mark in Figure 3 is average potentials. We found that there was a trend that the potential reproducibility deteriorated gradually with the decrease in *C*_L_. However, the deviation of each experimental potential data from the average potential in Figure 3 is not so large. Potential with the standard deviation was −141 ± 8 mV even when *C*_L_ was as low as 10^−5^ M. Another potential, when *C*_L_ was 3.4 M, was 72 ± 1 mV.

Although the discrepancy between the experimental and computational potential becomes larger at lower −log_10_([Cl^−^]_R_/[Cl^−^]_L_), they are still in good agreement with each other. As long as the concept of the GHK equation is valid, the discrepancy could be amended by considering the concentration of ions not taken into account for the potential computation of Figure 3. Namely, the KCl solution contains low level of protons and hydroxide. Those ions could affect on the potential generation, especially at the low level *C*_L_. Hereafter, the experimentally observed potential across the anion exchange membrane shown in Figure 3 is designated as *V*_aem_, and the computational data set shown in Figure 3 is designated as *V*_com_.

To sum up, these experimental results are in line with the prediction of GHK equation, and nothing unusual was observed. So, the experimental result in Figure 3 merely suggests the validity of the GHK equation. Hence, the experimental results provide no scientific significance at a glance. However, in the next section, some unexpected result causes a doubt about the validity of the GHK equation.

### 3.2. Potential Generation across the Ag Wire

#### 3.2.1. Experimental

Potential across the Ag wire generated by two KCl solutions was measured using the setup Figure 1b described in the Section 2.2 under the same environmental temperature and the same KCl solution conditions described in the Section 3.1. Experimental result (× mark) is shown in Figure 3, and hereafter this experimental data set is designated as *V*_Ag_. *V*_Ag_ behavior is quite similar to *V*_aem_ and even in better agreement with *V*_com_. Similarly to the procedure of potential measurements across the anion exchange membrane described in the previous section, once the potential reached the stable potential, experimental potential data was obtained. Compared with the potential measurement described in the previous section, the potential was quite stable and such stable potential was achieved much shorter period after supplying two KCl solutions in the left and right compartments of setup illustrated in Figure 1b. Measurements were carried out a number of times in order to assure the data reproducibility of potential. Hence, *V*_Ag_ in Figure 3 is average potential. There was a trend that the potential reproducibility deteriorated gradually with the decrease in *C*_L_, which is similar trend to the trend described in the previous section. However, the deviation of each experimental potential data from the average potential becomes less compared with those of the potential data *V*_aem_, and the deviation of most of the *V*_Ag_ from the average potential shown in Figure 3 is within the range of 6 mV. Potential with the standard deviation was −200 ± 6 mV even when *C*_L_ was as low as 10^−5^ M. Another potential, when *C*_L_ was 3.4 M was 91 ± 1 mV.

Surface of AgCl coated wire looks quite inhomogeneous as seen in Figure 2, but it did not affect the data reproducibility. We used a few AgCl coated wire, and none of them displayed significant alteration of potential compared with typical *V*_Ag_ shown in Figure 3.

Ag wire is undoubtedly impermeable to ions. However, the potential across the Ag wire was almost same as the potential across the anion exchange membrane which is permeable to ions. Is membrane permeability a primary factor for determining how high membrane potential is generated?

Ling and Cheng independently advocate their own but mutually similar theories for explaining the membrane potential generation mechanism [1,2,9,10,11,12,13]. Their theories basically attribute the origin of potential generation to the ion adsorption on a membrane or a separator which corresponds to the anion exchange membrane, an Ag wire and a glassy carbon plate in this work. They developed their theories applying the concept of adsorption isotherm. Following their basic ideas, we developed an analytical model for explaining the potential behavior of V_Ag_ considering ion adsorption on the surface of AgCl coat on the wire.

#### 3.2.2. Theoretical Analysis

**Figure 4:**
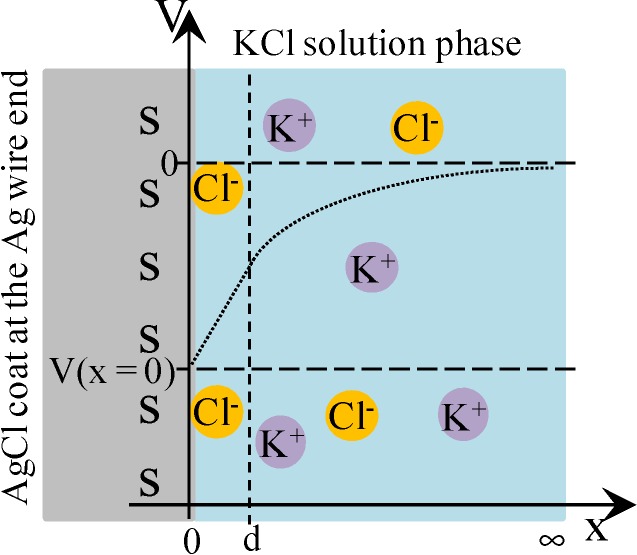
The interface between AgCl coat at the Ag wire end and KCl solution. *s* represents the adsorption site on the AgCl surface. Some chloride ions are in the adsorbed state on the AgCl surface. Coordinate system is set as illustrated, where *x* = 0 represents the interface plane between the AgCl surface and KCl solution phase and *x* = d represents the plane of ion diffuse phase closest to the adsorption site of AgCl. *V* represents the potential, where *V* = 0 at *x* = ∞. Dotted line represents the potential behavior expected.

Cheng reported that Cl^−^ adsorbs onto AgCl. Hence, we assume that AgCl at the ends of Ag wire illustrated in Figure 1b selectively adsorbs Cl^−^. Under this assumption, we derived an Equation explaining the potential behavior of *V*_Ag_. Figure 4 is an illustration at the interface between AgCl coat at the Ag wire end and KCl solution with a *V*,*x*-coordinate system, where *V* and *x* represent the potential and the distance from the adsorption site of AgCl surface, respectively. Some chloride ions are in the state of adsorption on the AgCl surface. Adsorption site s (see Figure 4) associates with Cl^−^. Hence, Equations (3)–(5) are derived using the concept of Langmuir isotherm, where K is an association constant.

*K* = 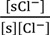
(3)

Total adsorption site concentration [s]_T_ is given by Equation (4).

[s]_T_ = [s] + [sCl^−^] (4)

Concentration of sCl^−^ is given by Equation (5) using Equations (3) and (4).

[sCl^−^] = 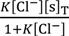
(5)


Charge density in KCl solution, ρ, is given by Equation (6) because of the Boltzmann distribution of ions.

ρ (*x*) = 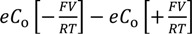
(6)
*e*: elementary charge, *C*_o_: concentration of K^+^ and Cl^−^ at *x* = ∞, *F*: Faraday constant, *V*: potential, *R*: gas constant, *T*: absolute temperature of experimental environment.

Owing to the charge neutrality, Equation (7) establishes.

−*e*[sCl^−^] + 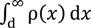 = 0
(7)
where *d* is the *x*-coordinate representing the plane in the ion diffuse phase closest to the adsorption site of AgCl (see Figure 4).

Employing Poisson-Boltzmann equation and Equation (6), Equation (8) is derived.

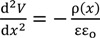
(8)
ε_o_: vacuum permittivity, ɛ: relative permittivity of water.

Equation (9) is derived by the use of Equations (5), (7) and (8), where the boundary condition d*V*/d*x* = 0 (*x*→∞) was applied.

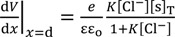
(9)


Equation (10) is derived by the use of Equations (6) and (8) under the boundary conditions of *V* = 0 (*x*→∞), d*V*/d*x* = 0 (*x*→∞), *V* < 0 (at any *x*) and d*V*/d*x* > 0 (at any *x*).

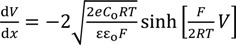
(10)


Solving Equation (10) with respect to *V* at *x* = d and the use of Equation (9) results in Equation (11).
*V* (*x* = d ) = 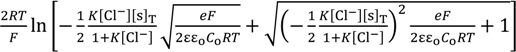
(11)


The association constant *K* usually relates the concentration of chloride ion at the bulk phase, [s] and [sCl^−^] one another. Hence, [Cl^−^] in Equation (11) can be given as *C*_o_. Therefore, Equation (11) is written as Equation (12).
*V* (*x* = d ) = 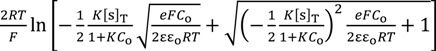
(12)


Electric field within the range 0 < *x* < d is expected to be constant of *E*_sur_. Hence, Equation (13) establishes.

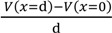
 = *E*_sur_(13)
where *E*_sur_ is given by Equation (14) owing to the Gauss’s law, and ɛ_low_ in the Equation (14) represents the permittivity of water within the range 0 < *x* < d. ɛ_low_ is expected to be much lower than ɛ due to the dielectric saturation of water [1].

−*E*_sur_ = 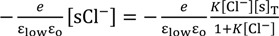
(14)


Equation (15) is derived by the use of Equations (9), (12)–(14).
*V* (*x* = 0) = 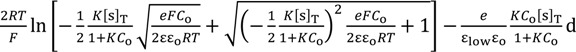
(15)
where *V*(*x* = 0) is graphically shown in Figure 4.

**Figure 5:**
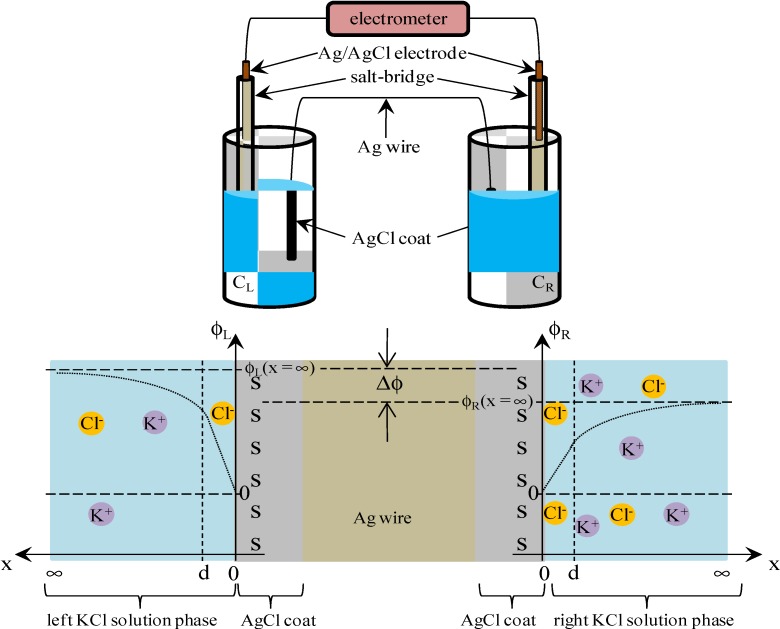
Graphical correspondence between experimental setup and adsorption model.


It is speculated that there exists nonzero potential in the bulk phase of KCl solution, when the potential at *x* = 0 was redefined 0 as illustrated in Figure 5. The nonzero potential in the KCl solution at *x* = ∞ in the left and right KCl solutions are redefined as ϕ_L_(*x* = ∞) and ϕ_R_(*x* = ∞), respectively, and the potential difference ∆ϕ defined by ∆ϕ = ϕ_L_(*x* = ∞) − ϕ_R_(*x* = ∞) corresponds to the potential we experimentally measured (see Figure 5). ϕ_L_(*x* = ∞) and ϕ_R_(*x* = ∞) are given by Equations (16) and (17), respectively.

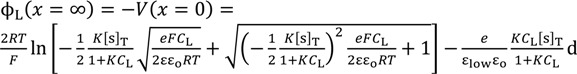
(16)


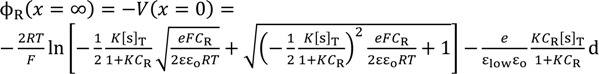
(17)

Substituting the Equations (16) and (17) into ∆ϕ = ϕ_L_(*x* = ∞) − ϕ_R_(*x* = ∞) gives us the theoretically expected potential based on the adsorption theory that the membrane potential is generated by the ion adsorption on the membrane (in concrete terms Ag wire separator) surface. By the use of Langmuir isotherm and Poisson-Boltzmann equation, the membrane potential is analytically given by ∆ϕ as so far described. Except for a quite small number of researchers like Drs. Ling and Cheng, almost no researchers have proposed the evaluation of membrane potential by employing such theories—Langmuir isotherm and Poisson-Boltzmann equation—instead of using GHK equation [1,2,9,10,11,12,13]. However, the basically same idea—potential evaluation by employing Langmuir isotherm and Poisson-Boltzmann equation—has been often used in the research fields outside the membrane potential research field for quantitatively estimating the potential behavior in electrolytic solution around charged surface [17,18,19,20]. Moreover, the quantitative potential evaluation around the charged surface in aqueous solution primarily using the Poisson-Boltzmann equation has been very common research for more than several decades [17,21,22,23,24,25,26,27,28,29,30,31,32]. Hence, the foundation of the theory the authors propose here has been already widely accepted and well discussed outside the research field of membrane potential for decades. However, such a well-established concept has never replaced the GHK equation.

**Figure 6:**
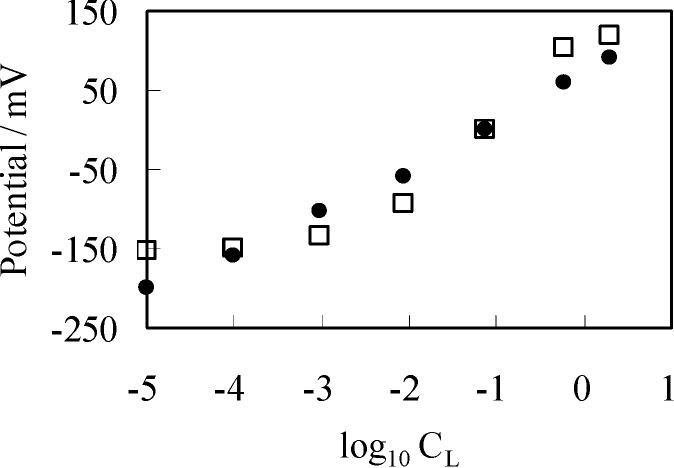
×: potential generated between two KCl solutions using a separator of Ag wire *vs.* log_10_*C*_L_ (=log_10_[Cl^−^]_L_); □: computational potential ∆ϕ based on the adsorption theory *vs.* log_10_*C*_L_ (=log_10_[Cl^−^]_L_); Data represented by • in this diagram is created by rearranging the data in Figure 3 represented by the same mark •.

As described in the Section 3.2.1, *V*_Ag_ was measured by increasing *C*_L_ from 10^−5^ M to 3.4 M, while maintaining *C*_R_ at 0.1 M. By the use of same condition, theoretically expected potential was obtained by computing ∆ϕ. For the computation, four factors should have been determined experimentally but were unable to be determined. They were [s]_T_, *K*, *d* and ɛ_low_. Hence, we assumed quite plausible quantities for them as follows: [s]_T_ was assumed to be 1.56 × 10^18^ m^−2^ by considering molecular dimension. It was guessed that K is quite low, since the experimentally obtained potential did not saturate by the increase of C_L_ even up to the highest concentration as seen in Figure 3. Thus, K was assumed to be 1 × 10^−25^ m^−3^. d was assumed to be 5 Å by considering interfacial structure illustrated in Figure 4. We could not find any decisive literature for determine ɛ_low_. However, some literature shows that hydrated ions permittivity is only a bit lower than the relative permittivity of water [33,34,35]. Hence, we chose 60 as ɛ_low_. The results are shown in Figure 6. Potential represented by × in Figure 6 was obtained by replotting the potential in Figure 3 represented by the mark × as a function of −log_10_*C*_L_. Computational potential represented by □ in Figure 6 represents ∆ϕ *vs.* log_10_*C*_L_ and the computational potential was obtained by considering ion activity of KCl [16], while the its horizontal axis quantities were all computed under the assumption that the activity coefficients were 1 just like Figure 3. Although the computational potential did not take into consideration the permeability of Ag wire to ions unlike the GHK equation, it well agrees with the experimental potential.

Although a good agreement between the experimental and computational potentials is seen in Figure 6, the adsorption theory has not been so strongly validated partially due to the assumption of some physical quantities used in the computation. We need to see if the adsorption theory is applicable to other experimental system. Hence, we further studied the adsorption theory as described in the following section.

### 3.3. Potential Generation across the Glassy Carbon Plate

#### 3.3.1. Experimental

**Figure 7:**
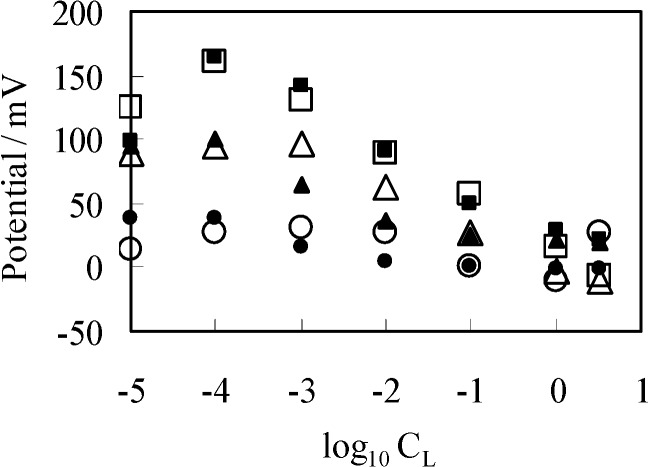
Potential generated between two KCl solutions using a separator of GC1 (GC2, GC3) *vs.* log_10_*C*_L_ (=log_10_[K^+^]_L_), where offset potentials are added to some data for decongesting the diagram. Open and closed marks represent the experimental and computational potentials, respectively. Circle marks: in case a GC1 used Triangle marks: in case a GC2 separator used (+25 mV offset potential is added to the actual data) Square marks: in case a GC3 separator used (+50 mV offset potential is added to the actual data).

Potential across the surface-modified glassy carbon plate separating two KCl solutions was measured using the setup Figure 1c described in the Section 2.3 under the same KCl solution conditions described in the Section 3.1, but environmental temperature was *T* = 301 K, not the same condition as in the previous experiments. Experimentally obtained potential is plotted against log_10_[K^+^]_L_ (=log_10_*C*_L_) as shown in Figure 7. Similarly to the procedure of potential measurements so far described, once the potential reached the stable potential, experimental potential data was obtained. Compared with the potential measurement described in the previous sections, it took a quite long time until the potential reached stable state. It took 4–6 h for achieving the stable potential after supplying two KCl solutions in the left and right compartments of setup illustrated in Figure 1c. The reason for such a slow realization of stable potential could be due to the rough surface of the surface-modified glassy carbon plate. Due to the rough surface of surface-modified glassy carbon plate, it took several hours until the ions migrated into the deep area of surface of the surface-modified glassy carbon plate. Because of such a slow process of stable potential generation, we were unable to obtain a large number of potential data which was statistically meaningful. Therefore, we paid careful attention to the experimental setup while carrying out the measurements. For example, we managed to prevent the solution evaporation from both left and right compartments of setup (Figure 1c), carefully handled the setup in order to prevent any mechanical impact on the experimental setup, monitored the environmental temperature carefully, and so on. Hereafter, the potential data set is designated as *V*_GC_. We take log_10_[K^+^]_L_ as *x*-axis of diagram in Figure 7 for the theoretical analysis of potential behavior to be described.

#### 3.3.2. Theoretical Analysis

**Figure 8:**
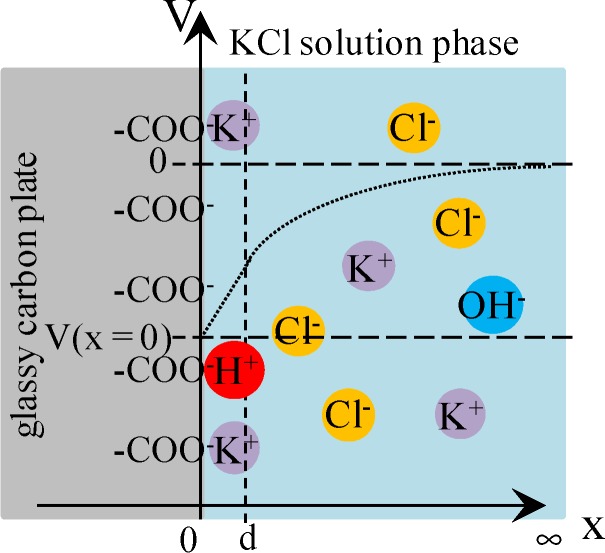
Interface between the surface-modified glassy carbon plate and KCl solution. Some potassium ions are in the state of adsorption to –COO^−^. Coordinate system is set in the same way as Figure 4.

Figure 8 illustrates the interface between the surface-modified glassy carbon plate and KCl solution. Some K^+^ and H^+^ are in the adsorbed state to –COO^−^. Equations (18) and (19) are derived for the dissociation of –COOH and that of –COOK, where *K*_h_ and *K*_k_ are dissociation constants for them, respectively.

*K*_h_ = 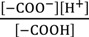
(18)

*K*_k_ = 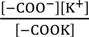
(19)

[–COO*X*]_T_ represents the total concentration of –COO^−^, –COOK and –COOH, and it is given by Equation (20).

[–COO*X*]_T_ = [–COO*^−^*] + [–COOH] + [–COOK] (20)

Equation (21) is derived by the use of Equations (18)–(20).

[–COO*^−^*] = 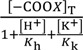
(21)


In the same manner as described in the Section 3.2.2, Equations (22) and (23) are derived.

ρ (*x*) = 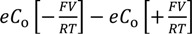
(22)

−*e* [–COO^−^] + 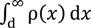 = 0
(23)


The same Poisson-Boltzmann equation expression as Equation (8) establishes for the system in question. By the use of the Poisson-Boltzmann equation, Equations (21) and (23), Equation (24) is derived as Equation (9) was derived, where the boundary condition d*V*/d*x* = 0 (*x*→∞) was employed.

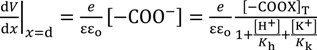
(24)


By the same procedure for deriving Equations (11), Equation (25) is derived.

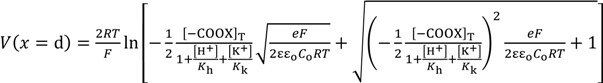
(25)


Employing Equation (24) and the same derivation procedure for Equations (16) and (17), Equations (26) and (27) are derived.

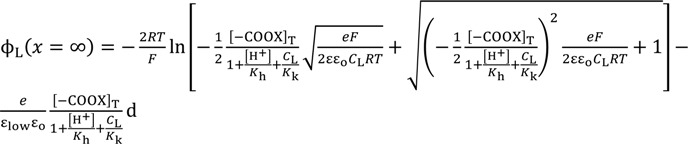
(26)

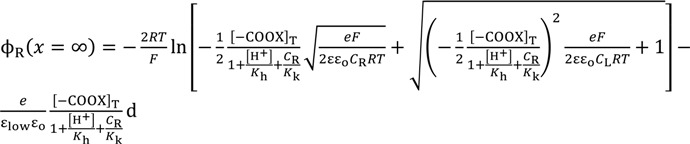
(27)


Again employing the same procedure described in the Section 3.2.2, the theoretically derived potential ∆ϕ for explaining the experimental potential behavior shown in Figure 7 is given by ∆ϕ = ϕ_L_(*x* = ∞) − ϕ_R_(*x* = ∞). Computational potentials are all given in Figure 7 along with the experimental data V_GC_. For the computation, [H^+^] was assumed to be 2.41 × 10^21^ m^−3^ (=4 × 10^−6^ M), since this experiment was carries out in the atmosphere. *K*_h_ was assumed to be 2.41 × 10^22^ m^−3^ (=4 × 10^−5^ M) based on the work described in the reference [32]. *K*_k_, *d*, ɛ_low_ and [–COO*X*]_T_ should have been determined experimentally but we could not determine them. Hence, we assumed quite plausible quantities for the first three as follows: Since –COOK is a salt, it is expected the dissociation constant of *K*_k_ is by far higher than *K*_h_. *K*_k_ was assumed to be 10-fold of *K*_h_, *K*_k_ = 2.41 × 10^22^ m^−3^. *d* was assumed to be 5 Å by considering the interfacial structure illustrated in Figure 8. We could not find any decisive literature for determining ɛ_low_. However, the relative permittivity of water around proteins is often assumed to be quite low for computer chemistry field or so [36]. It is possible to state one strong reason for that assumption: It is widely believed that water molecules surrounding protein form an ice-like structure and relative permittivity of ice is around 5 [37,38]. The surface-modified glassy carbon plate bears a number of carboxylic groups like proteins have. Hence, we chose 10 as ɛ_low_. As to [–COO*X*]_T_, the following quantities were assumed: [–COO*X*]_T_ = 1.38 × 10^16^ (≡[–COO*X*]_1_ for GC1), 4.00 × 10^16^ (≡[–COO*X*]_2_ for GC2), 25.0 × 10^16^ (≡[–COO*X*]_3_ for GC3) m^−2^. All the computationally obtained potentials relatively well reproduce the experimental results. According to the fabrication process of the surface-modified glassy carbon plates, it is expected that the density [–COO*X*] increases in the order of GC1–GC2–GC3. Indeed, we can see the relationship of [–COO*X*]_1_ < [–COO*X*]_2_ < [–COO*X*]_3_ as expected. The computational potential in Figure 7 was obtained by considering ion activity of KCl [16], while its horizontal axis quantities were all computed under the assumption that the activity coefficients were 1 just like Figure 3.

Experimental results *V*_GC_ exhibit unexpected behavior in low K^+^ concentration regime, that is, all the experimental diagrams have the highest peak of potential around at log_10_*C*_L_ = −4 or the highest plateau at and below log_10_*C*_L_ = −4. Even such unexpected potential behavior is reproducible theoretically. Hence, the adsorption theory as a generation mechanism for the membrane potential is strongly validated, although some physical quantities needed for the potential computation based on the adsorption theory were given by assumption. However, still one might emphasize that the potential across an impermeable membrane is generated by ion adsorption onto the membrane but the potential across a permeable membrane is generated by ion transport through the membrane. In order to defy this emphasis, we carried out another experiment shown in the next section.

### 3.4. Potential Generation across the Impermeable Ion Exchange Membrane

We made measurement of the potential across the impermeable ion exchange membrane. The procedure is described as below.

**Figure 9:**
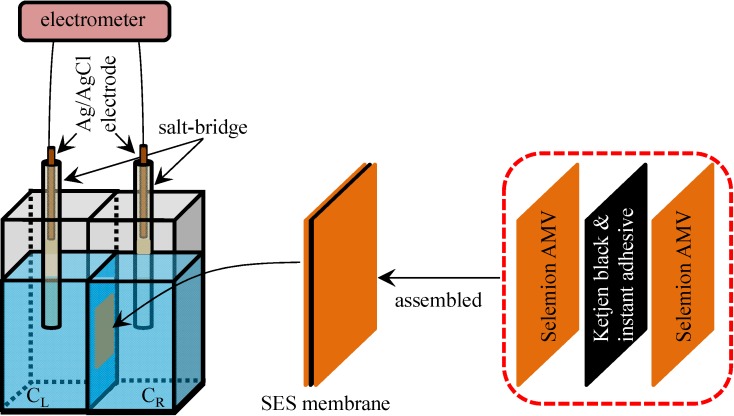
Structure of SES membrane and the experimental setup for measuring the potential across the SES membrane.

The impermeable ion exchange membrane was fabricated by gluing two sheets of Selemion AMV. As described in the Section 3.1, Selemion AMV is an anion exchange membrane and is quite permeable to anions but less permeable to cations, since it contains fixed cations and mobile anions in the hydrated state. Carbon called Ketjen black (Lion Corp., Tokyo, Japan), which is electrically highly conductive, was mixed with cyanoacrylate instant adhesive, resulting in an electrically conductive instant adhesive. Using this electrically conductive instant adhesive, two sheets of Selemion AMV was attached together. The resultant membrane was constituted of three layers: (Selemion AMV )-(electrically conductive adhesive)-(Selemion AMV) (hereafter called SES membrane). The electrically conductive adhesive layer of SES membrane is electrically conductive but impermeable to ions. Hence, the SES membrane is an impermeable ion exchange membrane. Potential across this SES membrane was measured following the exactly same procedure as described in the Section 3.1. The experimental setup and SES membrane structure are illustrated in Figure 9, The experimental setup and SES membrane structure are illustrated in Figure 9, and the setup is basically same as the setup illustrated in Figure 1a except for the type of membrane used.

**Figure 10:**
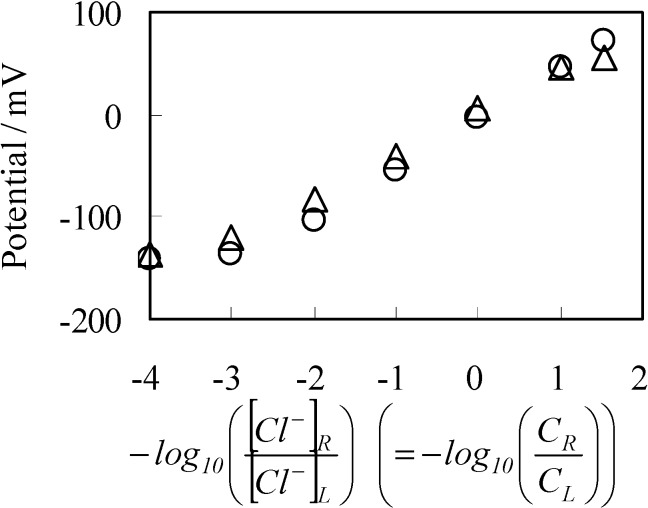
Potential generated between two KCl solutions *vs.* −log([Cl^−^]_R_/[Cl^−^]_L_). ○: Potential across the permeable membrane of Selemion AMV (same data as that shown in Figure 3); ∆: Potential across the impermeable membrane of SES membrane.

Figure 10 shows the potential across the SES membrane (impermeable membrane) along with the potential across the Selemion AMV (permeable membrane) which was earlier shown in Figure 3 with ○ mark. Both potentials across the SES membrane and Selemion AMV are virtually identical each other, although the former membrane is an impermeable membrane, while the latter one is a permeable membrane. Hence, it is natural to speculate that the membrane permeability does not play a central role for the membrane potential generation. The most plausible mechanism of membrane potential generation lies in the ion adsorption on to the membrane surface.

## 4. Conclusions

It was observed that the potential generated by two KCl solutions separated even by impermeable membrane (or separator) was almost the same as the membrane potential observed using an ion exchange membrane permeable to ions. This observation gave rise to a doubt as to the current concept of the membrane theory. Our experimental and theoretical analysis revealed that membrane potential behavior is explicable by adsorption theory instead of the GHK equation. The adsorption theory was capable of explaining the potential generation across the separators to the ions irrespective of their permeability to ions.

Based on the experimental and theoretical results, the authors believe that the origin of membrane potential so far believed explicable by GHK equation should be reconsidered and the GHK equation might be replaced with the adsorption theory.
